# Metagenomics reveals the effect of long-term fertilization on carbon cycle in the maize rhizosphere

**DOI:** 10.3389/fmicb.2023.1170214

**Published:** 2023-05-19

**Authors:** Yanan Li, Chengyu Wang, Hongyan Chang, Yumang Zhang, Shuxia Liu, Wentian He

**Affiliations:** ^1^College of Resources and Environment, Jilin Agricultural University, Changchun, China; ^2^Key Laboratory of Soil Resource Sustainable Utilization for Jilin Province Commodity Grain Bases, Jilin Agricultural University, Changchun, China; ^3^Institute of Plant Nutrition, Resources and Environment, Beijing Academy of Agriculture and Forestry Sciences, Beijing, China

**Keywords:** C fixation, C degradation, CH_4_ metabolism, long-term fertilization, metagenomics, maize rhizosphere soil

## Abstract

Long-term fertilization can result in the changes in carbon (C) cycle in the maize rhizosphere soil. However, there have been few reports on the impacts of microbial regulatory mechanisms on the C cycle in soil. In the study, we analyzed the response of functional genes that regulate the C fixation, decomposition and methane (CH_4_) metabolism in maize rhizosphere soil to different fertilization treatments using metagenomics analysis. As the dominant C fixation pathway in maize rhizosphere soil, the abundance of the functional genes regulating the reductive citrate cycle (rTCA cycle) including *korA, korB,* and *IHD1* was higher under the chemical nitrogen (N) fertilizer treatments [nitrogen fertilizer (N), compound chemical fertilization (NPK), the combination of compound chemical fertilizer with maize straw (NPKS)] than maize straw return treatments [maize straw return (S), the combination of phosphorus and potassium fertilizer with maize straw (PKS)]. The NPK treatment decreased the abundance of functional genes involved in 3-hydroxypropionate bicycle (3-HP cycle; *porA*, *porB*, and *porD*), which was one of the major C fixation pathways in soil aside from dicarboxylate-hydroxybutyrate (DC/4-HB cycle) and Calvin cycle. The abundance of functional genes related to C degradation was higher in S, PKS and NPKS treatments than N and NPK treatments, and chemical N fertilizer application had a significant effect on C degradation. The dominant Methanaogenesis pathway in maize rhizosphere soil, used acetate as a substrate, and was significantly promoted under chemical N fertilizer application. The functional genes that were related to CH_4_ oxidation (i.e., *pmoA* and *pmoB*) were reduced under N and NPK treatments. Moreover, soil chemical properties had a significant impact on the functional genes related to C fixation and degradation, with SOC (r^2^ = 0.79) and NO_3_^−^-N (r^2^ = 0.63) being the main regulators. These results implied that N fertilization rather than maize straw return had a greater influence on the C cycle in maize rhizosphere soil.

## 1. Introduction

In recent years, the essential role of soil microorganisms in regulating agricultural production and the carbon (C) cycle has been elucidated in numerous studies (Leadbeater et al., 2021; Ye et al., 2023). Yadav et al. (2021) proposed that soil microorganisms played significant roles in the ecosystem for nutrients cycling, and can be used for improving agro-environmental sustainability. Using microbiological systems to ameliorate agricultural production in a sustainable and eco-friendly way has been widely accepted as a future key-technology (Kumawat et al., 2022). Moreover, the structure and function of microbial community have an important impact on soil C emissions and are a major driver of the C cycle in rhizosphere micro-ecosystems, and the study of functional microbiomes has become a hot topic in recent years (Chen X. et al., 2023; Tang et al., 2023; Wu D. et al., 2023). Although the volume of rhizosphere soil is relatively small, due to the continuous secretion of various metabolites by plant roots and the discontinuous death and shedding of root surface tissues, the nutrients and microorganisms in rhizosphere soil are abundant, and their physical, chemical and biological properties are quite different from those in non-rhizosphere soil (Chen J.-Z. et al., 2023). And Cai et al. (2022) confirmed that the rhizosphere is the most active soil area, which plays an important role in soil biochemical cycling. Specially, the composition and function of the microorganisms in rhizosphere soil has drawn more attention due to the important role that the rhizosphere soil played in the uptake of mineral nutrients and water by plants (Castellano-Hinojosa et al., 2021). And soil functional microbiome is crucial to understand the ecological processes and functions such as nutrient cycles, conversion of C substances into carbon dioxide (Enebe and Babalola, 2022). The changes in the abundance of genes related to C metabolism and microbial community structure could lead to the differences in C metabolism functions, which would further affect the dynamics of soil “C source” and “C sink” in farmland ecosystem (Deng et al., 2019; Wang J. et al., 2021). Hence, the study of soil microorganisms could provide essential new insights into the regulatory mechanism of the C cycle in rhizosphere soils.

Long-term fertilization, which adds nutrients into agroecosystems to improve crop production to meet the growing food demands, is a common phenomenon in China at present (Li et al., 2009; Gao et al., 2023). However, long-term fertilization application, especially excessive fertilization could cause a series of environmental problems, such as soil acidification and greenhouse gas (GHG) emissions (Jin et al., 2022). Maize straw is abundant in organic matter, nitrogen (N), phosphorus, potassium and other nutrients, and could be used as renewable organic fertilizer (Cheng et al., 2018). Previous studies confirmed that long-term fertilization had a significant impact on the stability of soil C carbon and C emissions (Zhang et al., 2021). According to previous reports, soil physicochemical properties played important roles in controlling microbial community structure (Lauber et al., 2008; Val-Moraes et al., 2016). In addition, many bacterial communities are closely correlated with soil chemical properties and can be used as indicators of soil condition (Kuramae et al., 2015). Investigating the correlation between soil microbial communities and soil chemical properties can enable a better understanding of the mechanism of soil C cycle. However, the mechanism of soil C cycling under different fertilizer application is still unclear. Therefore, more attention should be paid to explore the association and underlying mechanism of long-term fertilization for regulating soil C cycle.

Fertilization has a significant effect on the dynamical balance of the soil C cycle (B. Liu et al., 2022). Hu et al. (2022) found that soil C fixation and degradation processes were significantly affected by manure application, but were not significantly changed under chemical fertilizer application. Fertilizer application promotes soil carbon dioxide (CO_2_) emissions by increasing the abundance of catabolic genes involved in carbon cycling (Enebe and Babalola, 2021). Maize straw is an important source of organic C in agroecosystem and can substantially increase the soil carbon stock (Chen et al., 2022). Furthermore, chemical N fertilization increased the abundance of methanogenic genes in croplands (Yuan et al., 2018). And the magnitude of the effect of fertilizer application on soil microbiome is depended on the soil properties such as soil N and C contents and fertilizer source (Castellano-Hinojosa et al., 2021). Recently, the studies investigating the soil functional microbial communities were mainly focused on the abundance of functional genes. However, the impacts of different long-term fertilization management on the microbial function genes relate to C cycling processes need to be determined.

In this study, the metagenomic sequencing technology was applied to quantify the C metabolic pathways (including metabolic modules and the functional genes related to C cycle) and analyze the potential metabolic response to long-term fertilizers application. We aimed to (i) explore the effects of long-term fertilizer application on soil C cycle processes at the level of metabolic modules and functional genes. (ii) To analyze the correlation between the soil properties and the abundance of functional genes related to C cycle in maize rhizosphere soil. This study hypothesized that the functional genes related to C cycle will be altered in maize rhizosphere under long-term fertilization.

## 2. Materials and methods

### 2.1. Experimental design

The rhizosphere soil samples were collected from a long-term experimental site located in Jilin Agricultural University, Changchun City, Jilin Province, Northeast China, which has a semi-humid temperate continental climate (43°47′42″ N, 123°20′45″ E). The maize field experiment was established in 1984, with the maize seeds sown in April, and all maize residues were removed from the plot after harvest in October. The maize was rained without additional irrigation. We collected the rhizosphere soil in polyethylene bags by shaking the roots until the non-adhering soil fell off. Soil samples were collected after the harvest period, each plot was sampled at five points, and the samples were mixed into one sample for a total of 18 test samples. Six treatments, including no fertilization (CK), N fertilization (N, 150 kg/ha), compound chemical fertilization (NPK, N-P-K: 150–75-75 kg/ha), maize straw return (S, 5,000 kg/ha), the combination of phosphorus and potassium fertilizer with maize straw (PKS, P-K-S: 75-75-5,000 kg/ha), the combination of compound chemical fertilizer with maize straw (NPKS, N-P-K-S: 150-75-75-5,000 kg/ha), were implemented with three replicates per treatment for this study.

The methods adopted for the measurements of soil properties, i.e., soil pH, total N (TN), total phosphorus (TP), total potassium (TK), ammonium-N (NH_4_^+^-N), nitrate-N (NO_3_^−^-N), available phosphorus (AP) and available potassium (AK) were described in Li et al. (2020). The readily oxidizable organic carbon (ROC) was determined by potassium permanganate oxidation method, and dissolved organic carbon (DOC) was determined by TOC analyzer (Huang et al., 2021). The measured soil properties were listed in Supplementary Table S1.

### 2.2. Metagenomics sequencing, contig assembly, and annotation

The microbial DNA extraction and metagenomic sequencing was conducted according to previous research (Li et al., 2020). The non-redundant contigs set is obtained by using the MMseqs2 software. The (merged) contigs sequence set is de-redundant according to the similarity degree of 95% and the coverage degree of alignment area of 90%. Then the contigs sequence set were further filtered to remove nontarget fragment sequences and sequences with an actual depth of 0. Subsequently, contigs were used for the prediction of open reading frames (ORFs) using MetaGeneMark,^1^ then translated into amino acid sequences. The non-redundant gene catalog was searched against Kyoto Encyclopedia of Genes and Genomes^2^ for functional annotation. A total of 1,605,676,212 reads were obtained after sequencing, of which 51,418,797 contigs sequences were retained after filtering and removing chimeras (Supplementary Table S2).

### 2.3. Statistical analyses

We used analysis of variance (ANOVA) to analyze the differences among means, followed by least significant difference (LSD) test if the difference was significant. Redundancy analysis (RDA) was used to analyze the contribution of environmental factors (soil properties) to the changes in functional genes related to soil C cycle. Principal components analysis (PCA) was used to identify the effect of fertilizer application on the abundance of functional genes related to C cycle. The RDA and PCA analysis were performed by a free online platform for data analysis.^3^ We used Spearman’s rank correlation coefficients to examine the associations between the functional genes related to C cycle and soil properties, and illustrated heatmaps to show the results by an online tool of Majorbio Cloud Platform.^4^

## 3. Results

### 3.1. Carbon metabolic pathways in soil

**Figure 1:**
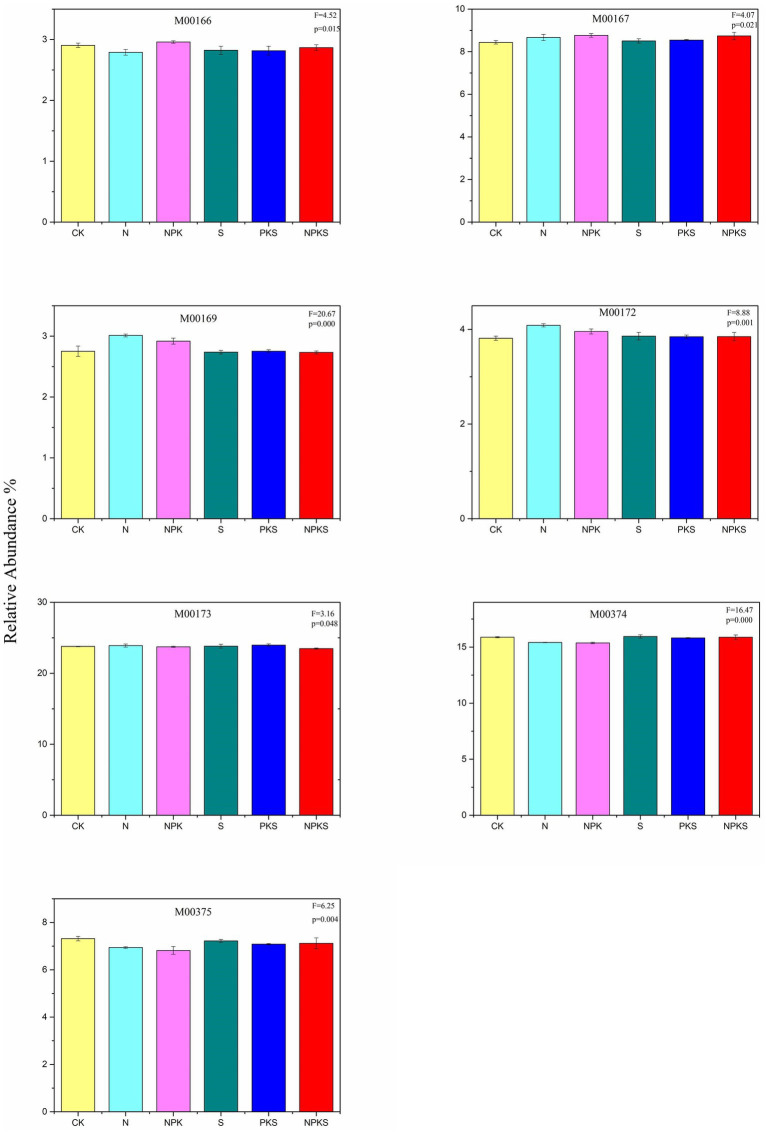
The relative abundance of CO_2_ fixation pathways (the significance test at *p* < 0.05 level) in maize rhizosphere soils under different fertilizer applications. Reductive pentose phosphate cycle (M00165, Calvin cycle; M00166, ribulose-5P = > glyceraldehyde-3P; M00167, glyceraldehyde-3P = > ribulose-5P), CAM cycle (M00168, dark; M00169, light), C4-Dicarboxylic acid cycle (M00170, M00171, M00172), rTCA cycle (M00173), DC/4-HB cycle (M00374), 3-HP/4-HB cycle (M00375), 3-HP cycle (M00376), and Wood-Ljungdahl pathway (M00377).

There were a total of 164 genes participated in the soil C cycle according to the KEGG database in maize rhizosphere soil. The reductive citrate cycle (rTCA) was the dominant C metabolic pathway in maize rhizosphere soil (38 genes, 23.48–23.97%), and its relative abundance was changed significantly under different treatments (*p* < 0.05). The following was the dicarboxylate-hydroxybutyrate (DC/4-HB cycle, 20 genes, 15.37–15.95%), and N and NPK treatments significantly decreased its reductive abundance (*p* < 0.001). There were 21 genes participating in the Calvin cycle (12.24–12.51%) and 3-hydroxypropionate bicycle (3-HP cycle, 13.03–13.30%), and their relative abundance was not significantly different from each other under different fertilization treatments (*p* > 0.05). Only a small number of genes participated in the Crassulacean acid metabolism (CAM) cycle and C_4_-Dicarboxylic acid cycle, and the relative abundance of which increased significantly under N and NPK treatments (M00169, M00172, *p* < 0.001). Chemical fertilizer (N and NPK treatments) and NPKS treatments increased the abundance of glyceraldehyde-3P = > ribulose-5P (M00167). Furthermore, S and PKS treatments had no significant impact on C cycle at the module level compared with CK treatment except for M00375 (Figure 1; Supplementary Figure S1).

**Figure 2:**
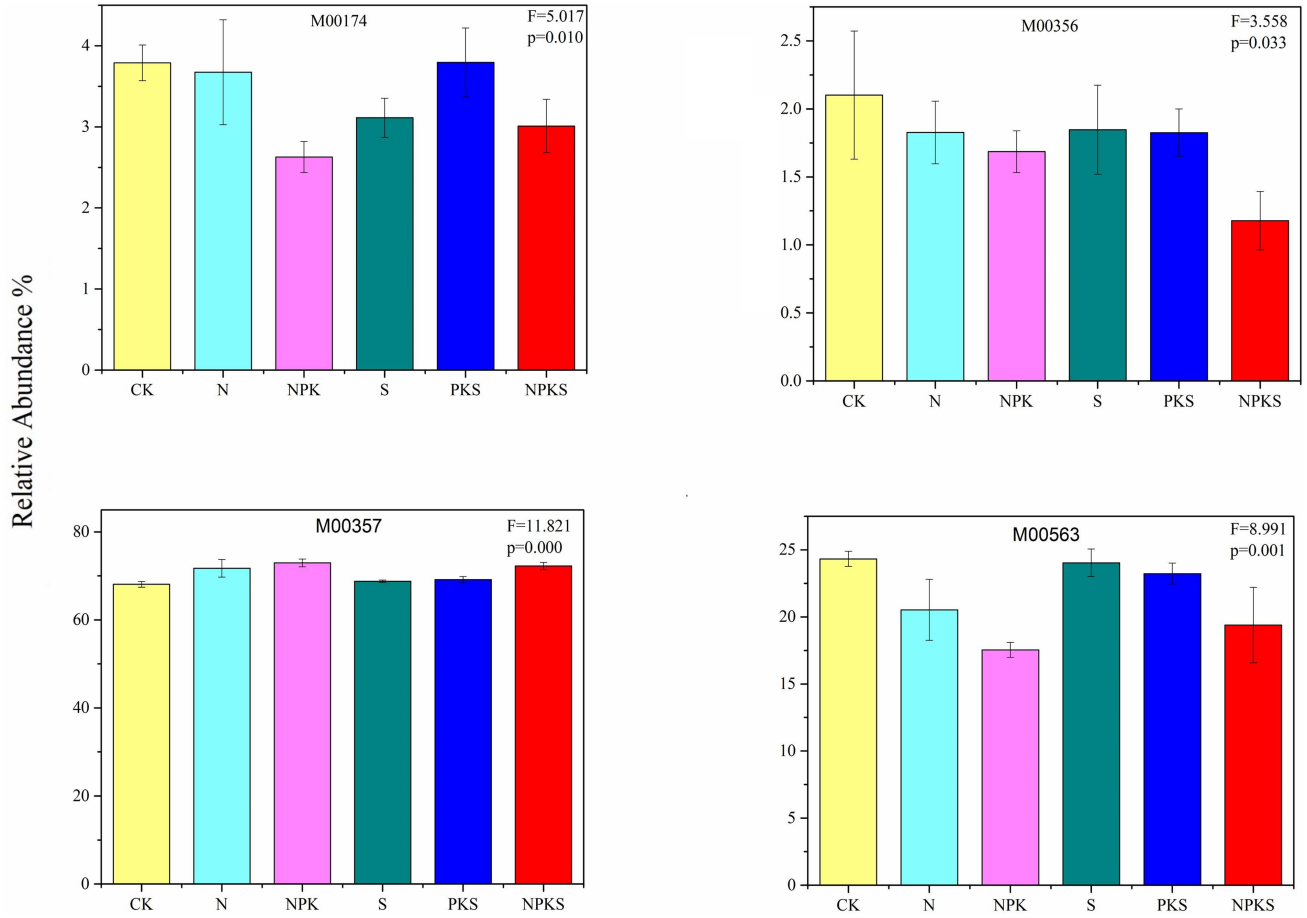
The relative abundance of methane metabolic pathways (the significance test at *p* < 0.05 level) in maize rhizosphere soils under different fertilizer applications. Methane oxidation: M00174, Methanaogenesis: methanol = > methane (M00356), acetate = > methane (M00357), methylamine/dimethylamine/trimethylamine = > methane (M00563).

A total of 70 genes were involved in the methane (CH_4_) metabolic pathways. 11 genes participated in CH_4_ oxidation (M00174), and compared with CK treatment, the relative abundance of CH_4_ oxidation pathway had significantly changed under fertilization treatments except for N and PKS treatment. The relative abundance of CH_4_-producing metabolic pathways that used acetate as a substrate was the highest in maize rhizosphere soils (68.08–72.94%), and increased significantly under N fertilizer application (*p* < 0.001). In contrast, N application significantly decreased the relative abundance of CH_4_-producing metabolic pathways that used methylamine, dimethylamine and trimethylamine as substrates (*p* < 0.001). Furthermore, 19 genes participated in CH_4_-producing metabolic pathways that use CO_2_ as a substrate, and its relative abundance did not significantly change under different fertilizer application treatments (*p* > 0.05; Figure 2; Supplementary Figure S2).

### 3.2. Functional genes involved in C cycle in soil

A subset of 53 genes involved in C cycling was retrieved from the 18 metagenomes, all genes listed in Supplementary Table S3. They were involved in C fixation and degradation and CH_4_ metabolism (Wang S. et al., 2021). The functional genes related to C fixation exhibited the highest abundance, followed by the C degradation functional genes (Table 1). Within the C fixation group, the relative abundance of functional genes related to multiple systems (13.76–15.36%), C monoxide (CO) oxidation (18.34–21.15%), and the rTCA cycle (13.58–15.37%) was higher than that of functional genes participated in the other C fixation pathways. Within the C degradation group, functional genes related to hemicellulose degradation exhibited highest abundance (9.29–10.98%) across all treatments, followed by starch degradation (7.49–8.13%) and cellulose degradation (3.20–4.33%) genes.

**Table 1:** The relative abundance of functional genes participated in carbon cycle in maize rhizosphere soils under different fertilizers application.

	CK	N	NPK	S	PKS	NPKS
Methanogeneiss	2.06 ± 0.07a	1.55 ± 0.23b	1.34 ± 0.04b	2.10 ± 0.04a	1.96 ± 0.04a	1.59 ± 0.26b
Methane oxidation	0.35 ± 0.03a	0.31 ± 0.06ab	0.22 ± 0.02c	0.27 ± 0.02bc	0.32 ± 0.03ab	0.22 ± 0.02c
CO oxidation	20.95 ± 0.56a	21.15 ± 0.70a	18.35 ± 0.90c	21.10 ± 0.72a	20.29 ± 0.25ab	19.49 ± 0.35b
Calvin cycle	0.66 ± 0.11a	0.65 ± 0.05a	0.72 ± 0.07a	0.67 ± 0.10a	0.60 ± 0.01a	0.68 ± 0.00a
Reductive acetyl-CoA pathway	2.09 ± 0.08a	2.06 ± 0.09a	1.95 ± 0.07a	2.12 ± 0.07a	2.07 ± 0.17a	2.07 ± 0.08a
rTCA cycle	13.76 ± 0.48b	15.04 ± 0.28a	15.37 ± 0.26a	13.58 ± 0.50b	13.58 ± 0.28b	13.98 ± 0.52b
Multiple systems	28.60 ± 0.38c	29.38 ± 0.61b	30.22 ± 0.47a	28.36 ± 0.37c	28.13 ± 0.30c	29.43 ± 0.22b
3HP cycle	4.70 ± 0.36a	4.79 ± 0.43a	3.82 ± 0.16b	4.52 ± 0.18a	4.67 ± 0.09a	3.62 ± 0.10b
DHDC/4-HB cycle	3.75 ± 0.13a	3.76 ± 0.14a	3.76 ± 0.10a	3.93 ± 0.14a	3.84 ± 0.14a	3.97 ± 0.23a
Hemicellulose	10.71 ± 0.15a	9.29 ± 0.16c	9.93 ± 0.16b	10.93 ± 0.37a	10.98 ± 0.13a	10.68 ± 0.43a
Chition	1.13 ± 0.07a	1.14 ± 0.04a	1.35 ± 0.11a	1.15 ± 0.03a	1.25 ± 0.09a	1.24 ± 0.13a
Cellulose	3.22 ± 0.03b	3.20 ± 0.04b	4.33 ± 0.22a	3.39 ± 0.24b	3.59 ± 0.32b	4.29 ± 0.44a
Pectin	0.28 ± 0.03bc	0.24 ± 0.03c	0.35 ± 0.04abc	0.30 ± 0.03abc	0.39 ± 0.14bc	0.41 ± 0.07a
Starch	7.74 ± 0.16b	7.49 ± 0.00c	8.11 ± 0.07a	7.41 ± 0.05c	8.02 ± 0.03a	8.13 ± 0.12a

**Figure 3:**
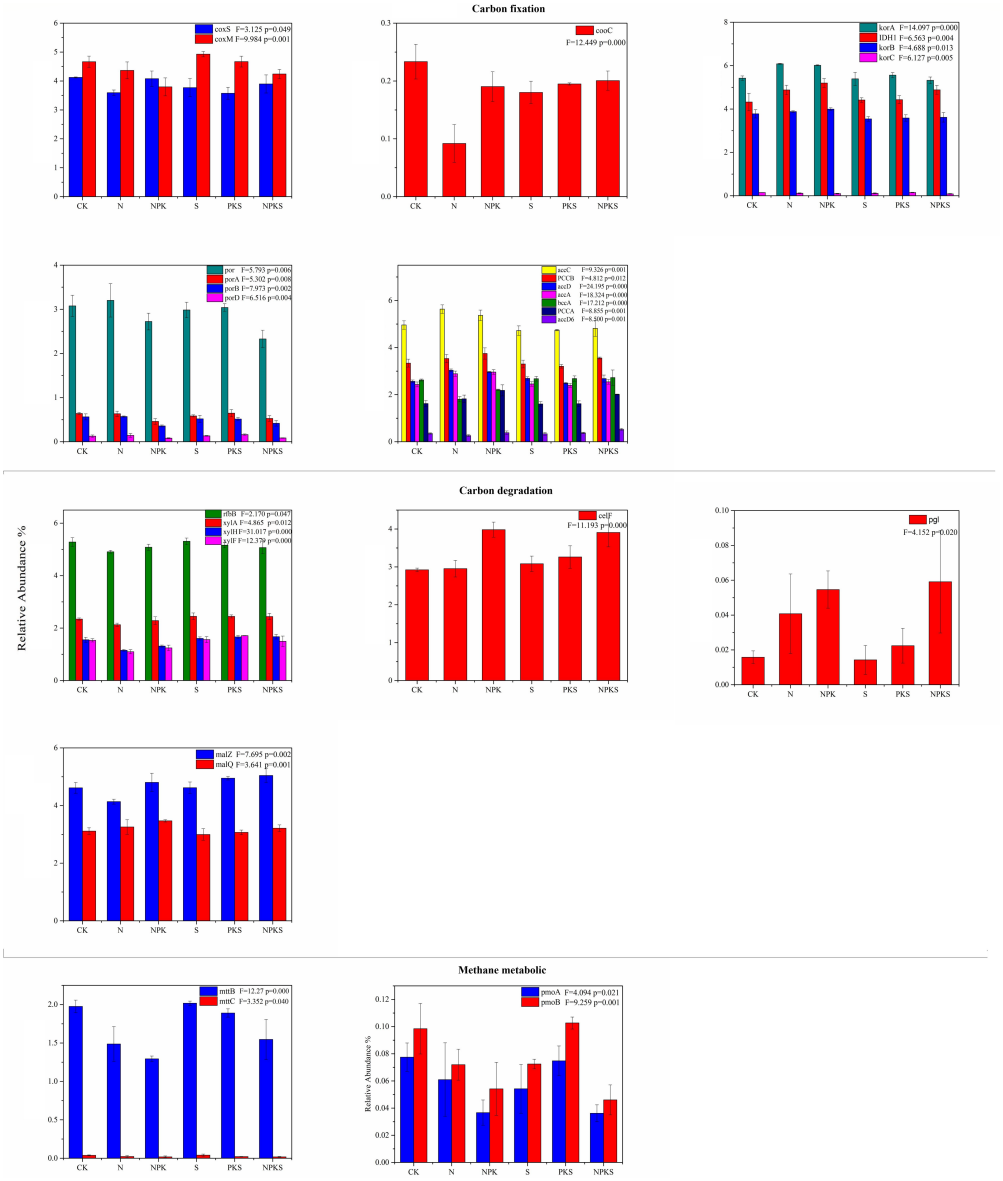
The relative abundance of functional genes participated in carbon cycle (the significance test at *p* < 0.05 level) in maize rhizosphere soils under different fertilizer applications. *coxS* and *coxM* involved in carbon-monoxide oxidation; *cooC* involved in reductive acetyl-CoA pathway; *korA*, *korB*, *korC*, and *IDH1* involved in the rTCA cycle; *porA*, *porB*, *porD*, and *por* involved in 3HP cycle; *bccA*, *PCCA*, *PCCB*, *accD6*, *accA*, *accC*. and *accD* involved in multiple systems in carbon fixation. In carbon degradation *xylA*, *xylF*, *xylH*, and *rfbB* involved in hemicellulose; *celF* involved in cellulose; *pgl* involved in pectin; *malZ* and *malQ* involved in starch. In methane metabolic *mttB* and *mttC* involved in methanogenesis; *pmoA*, *pmoB*, involved in methane oxidation.

Functional genes participated in CO oxidation (*coxS*) were decreased significantly under N treatment (*p* < 0.05), similar with the effects of fertilizer application on *cooC* gene related to reductive acetyl-CoA pathway. In the rTCA cycle, the relative abundances of *korA*, *korB* and *IHD1* were higher under chemical fertilization treatments than maize straw return treatments. The NPK treatment decreased the functional genes involved in 3-HP cycle (*porA*, *porB*, and *porD*). No significant changes were found in the functional genes related to Calvin cycle and DC/4-HB cycle under different fertilizer application treatments (*p* > 0.05). The relative abundance of *PCCA*, *PCCB*, *accA*, *accC*, and *accD* related to multiple system in C fixation was also markedly enhanced by the N, NPK and NPKS treatments (Figure 3).

Compared with CK treatment, N and NPK treatment significantly decreased the relative abundance of functional genes (including *xylA*, *xylH*, *xylF*, and *rfbB*) related to hemicellulose degradation (*p* < 0.05). However, other treatments had no significant effects on hemicellulose degradation genes (*p* > 0.05). Besides, the relative abundance of the functional genes related to cellulose (*celF*) and pectin (*pgl*) for NPK and NPKS treatments were higher than that for other treatments. The abundance of starch degradation gene *malZ* declined under N fertilizer application, increased under NPKS fertilizer application (*p* < 0.05). The N, NPK, and NPKS treatments inhibited the process of methanogenesis. Compared with N fertilizer applications, the relative abundance of *mttB*, which participated in methanogenesis, was the higher under straw return application. Moreover, the N, NPK, and NPKS treatments reduced the abundance of CH_4_ oxidation genes in maize rhizosphere soil (Figure 3).

### 3.3. Correlations between microbial functional genes and soil properties

**Figure 4:**
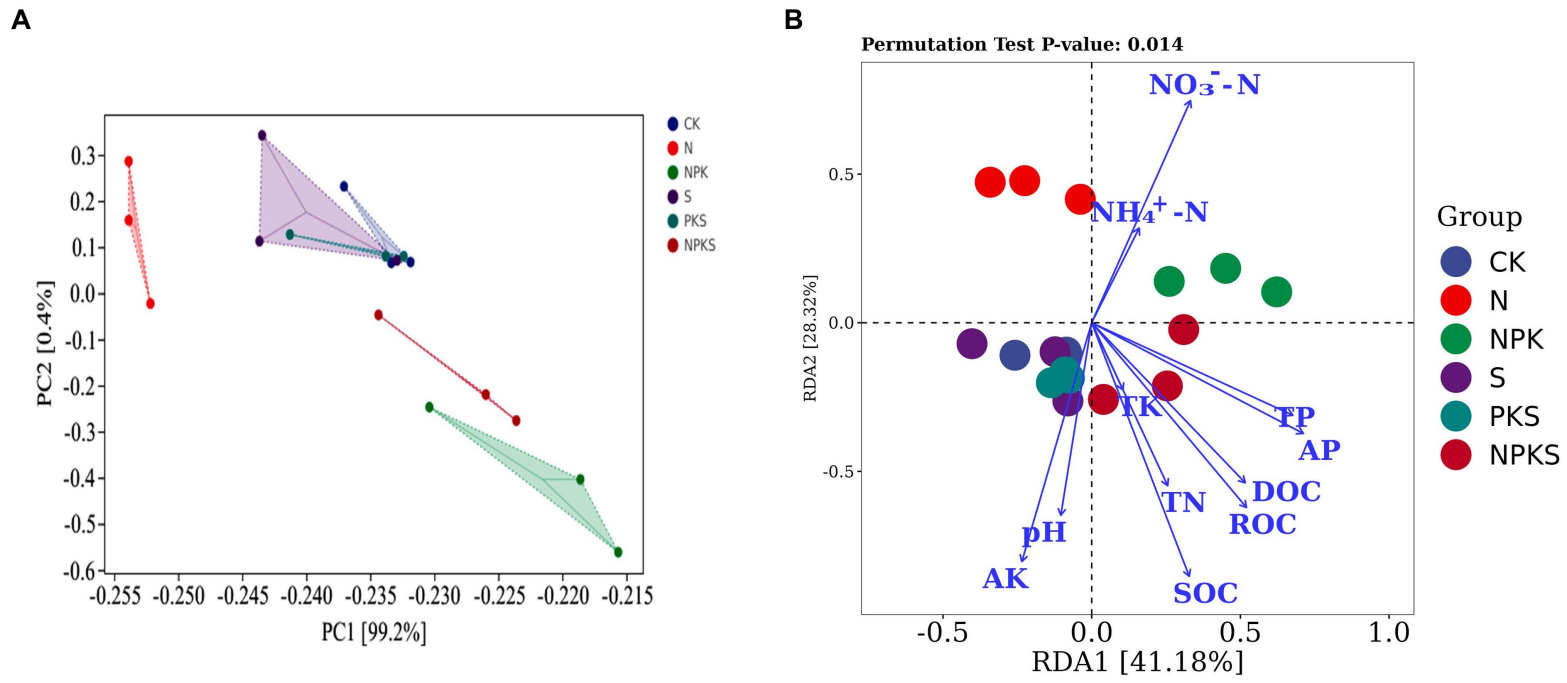
Effects of fertilizers application on functional genes in soil carbon cycles. Functional genes identified by principal components analysis **(A)**. Redundancy analysis of microbial functional genes associated with soil properties **(B)**. SOC, soil organic carbon; DOC, dissolved organic carbon; ROC, readily oxidizable organic carbon; TN, total nitrogen; TP, total phosphorus; TK, total potassium; NH_4_^+^-N, ammonium nitrogen; NO_3_^−^-N, nitrate nitrogen; AP, active phosphorus; AK, active potassium.

**Figure 5:**
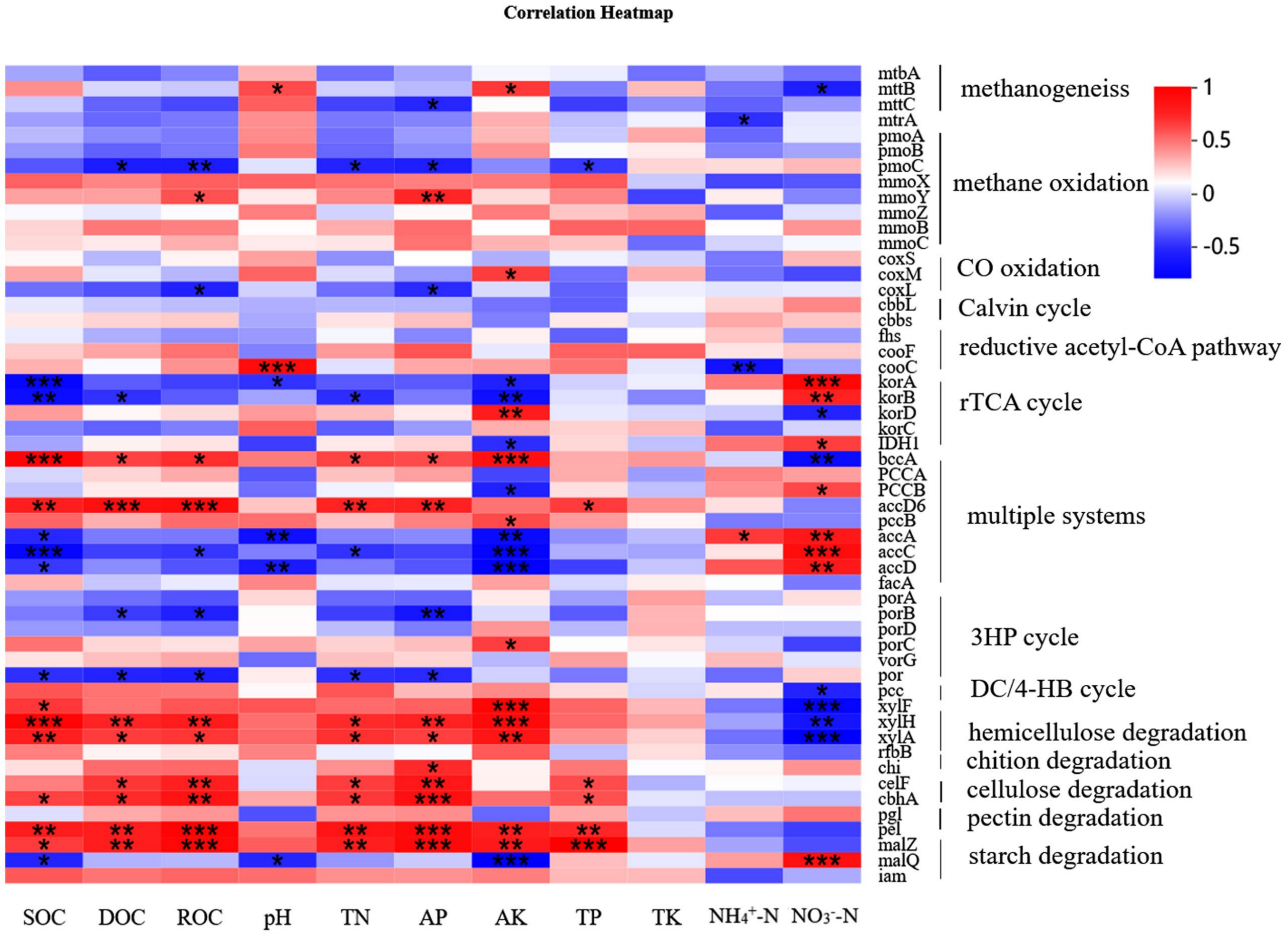
The heatmap of the correlation between microbial functional genes and soil properties.

The results of PCA indicated that fertilizer application explained most of the variation in the C cycling genes across all samples (PC1 of PCA: 99.2%). The sample points from the CK-, S- and PKS-treated soils grouped together and were separated from those of other treatments (Figure 4A). The variations among these genes under different treatments were probably caused by the soil properties. The RDA showed that the different forms of soil C (soil organic carbon [SOC], dissolved organic carbon [DOC], readily oxidizable organic carbon [ROC]) and available nutrients (nitrate N [NO_3_^−^-N], active potassium [AK], active phosphorus [AP], total phosphorus [TP]) and pH had significant effects on C cycle (*p* < 0.05; Figure 4B). The soil properties had strong effects on the C fixation pathways including reductive acetyl-CoA pathway (*cooC*), rTCA cycle (*korA*, *korB*, and *IDH1*), 3HP (*porB*) and multiple systems (*bccA*, *accA*, *accC*, and *accD*). For C degradation, SOC, DOC, ROC, total nitrogen (TN), AP and AK were significantly positively correlated with the hemicellulose (*xylF*, *xylH*, and *xylA*) and cellulose (*celF* and *cbhA*) and pectin (*pel*) degradation. The NO_3_^−^-N was significantly negatively correlated with *xylF*, *xylH*, and *xylA*, which were relative with hemicellulose degradation, and was positively correlated with starch degradation gene *malQ*. However, the soil properties had no significant correlation with the functional genes related to the Calvin cycle. Moreover, the methanogenesis-relative genes *mttB* had positive correlation with pH and AK, and negative correlation with TN, ammonium nitrogen (NH_4_^+^-N) and NO_3_^−^-N. Functional gene *pmoC* that participated in CH_4_ oxidation had strong correlation with DOC, ROC, TN, AP, and TP (Figure 5; Supplementary Table S4).

## 4. Discussion

### 4.1. Effects of fertilizer application on soil C fixation and CH_4_ metabolism at the module level

Fertilizer application could influence soil C turnover (Gong et al., 2012). Similarly, in this study, chemical fertilizer application decreased the SOC content, while maize straw return and chemical fertilizer added with maize straw increased the SOC content compared to CK treatment. Active organic carbon is an essential component of soil organic carbon, the increase of its content can provide more available carbon sources for soil microorganisms, and then affect soil carbon cycle (Luo et al., 2018). Moreover, fertilizer application stimulated the transformation of stable SOC to labile forms such as DOC and ROC (Supplementary Table S1). The result confirmed that long-term fertilization can affect the C cycle by changing the content of different forms of soil carbon. Soil C transformation was found to be driven by microorganisms (Kramer and Gleixner, 2006) and previous studies have shown that soil microorganisms participate in the transformation of various forms of C in soil, and can mediate the CO_2_ fixation and C degradation (Beulig et al., 2016; Agarwal et al., 2017; Jacoby et al., 2017). This study assessed eight CO_2_ fixation pathways including the plant photosynthesis pathway (CAM cycle) in maize rhizosphere soil that existed in the KEGG database (Li et al., 2022). It might be because the maize rhizosphere could provide the substrates needed for microbial photosynthesis. As the dominant C fixation pathway, the rTCA cycle only needed two adenosine triphosphates (ATP) to complete this metabolic process (Claassens et al., 2016), and the combined application of chemical fertilizer and maize straw (NPKS) decreased the abundance of the rTCA (Figure 1). Compared to the rTCA cycle, the Calvin cycle, a major pathway for CO_2_ fixation that catalyzes the carboxylation of ribulose-1,5-bisphosphate into two molecules of 3-phosphoglycerate, requires more energy (Huang et al., 2022). Long-term fertilization could affect C cycle by altering C metabolism pathways. It was reported that long-term fertilizer application had no significant effects on the Calvin cycle (Hu et al., 2022), but the glyceraldehyde-3P = > ribulose-5P (M00167) metabolic pathway was strongly improved under chemical fertilization (N and NPK treatments) and the combined application of chemical fertilizer and maize straw (NPKS) in this study (Figure 1). Previous study concluded that soil water content and N fertilizer had significant effects on the CAM, and N fertilizer changed the net CO_2_ uptake and photosynthetic capacity of the CAM in soil (Ma et al., 2022). Similar results were observed in this study, that is, compared with CK treatment, chemical fertilization (N and NPK treatments) promoted the CAM metabolic pathway. In addition, chemical fertilization also increased the relative abundance of the C4-Dicarboxylic acid metabolic pathway. Moreover, compared with the CK treatment, maize straw return and the combined application of maize straw and PK fertilizer had no significant effects on C fixation metabolic pathways, indicating that the C fixation in soil was mainly affected by chemical N fertilizers.

Among the different agricultural production systems, paddy fields are a source of CH_4_ emissions whereas dryland is a weak sink for CH_4_ uptake (Snyder et al., 2009; Wu P. et al., 2023). Previous studies mainly focused on CH_4_ emissions, whereas the microbial process of CH_4_ metabolism in soil was less understood. Methane metabolism mainly consists of methanaogenesis and CH_4_ oxidation that occurred in maize rhizosphere soil. The dominant metabolic pathways of methanaogenesis used acetate as the substrate (Padhy et al., 2022), thus increasing significantly under chemical N fertilization and the combined application of chemical N fertilizer with maize straw. Oppositely, the chemical N fertilization inhibited the methanaogenesis pathway from methylamine, dimethylamine and trimethylamine. Methanosphaera could reduce the methyl group of methanol to CH_4_ (Ferry, 2010), the abundance of which decreased under chemical fertilizer application and combined application of chemical fertilizer and maize straw (Supplementary Figure S3). Moreover, chemical fertilization and maize straw return had no significant effect on CH_4_ metabolism that utilized CO_2_ as a substrate, probably because the Methanocella genera grow and produce CH_4_ from CO_2_ and H_2_, which was not significantly affected by N fertilization treatments (Yuan et al., 2018; Supplementary Figure S3).

### 4.2. Mechanisms of fertilizer application for regulating soil C cycle

The functional genes related to C fixation have been studied extensively in terrestrial ecosystems and most of the previous studies focused on the *cbbL* and *cbbM* genes driving the Calvin cycle (Liu et al., 2016; Li et al., 2017; Treseder et al., 2018). However, we found the relative abundance of *cbbL* and *cbbM* genes was lower than the functional genes participated in multiple system, CO oxidation and rTCA cycle. This result is similar to the pattern of soil C metabolism at module level. Chemical N addition can induce a shift in soil N availability, which influences soil C turnover (Wang et al., 2018). In this study, the abundances of the functional genes related to C fixation including the rTCA cycle, 3HP cycle, multiple system and CO oxidation responded distinctively to different fertilization treatments. This was similar with (Schleuss et al., 2019), but different from the results reported by Hu et al. (2022), which was probably because the samples were obtained from the maize rhizosphere soil in this study and surface soil in previous study, respectively. Three functional genes related with the CO oxidation process were obtained from the maize rhizosphere, among which the abundance of *coxL* was the highest and did not significantly vary among different treatments. However, the abundance of *coxS* and *coxM* decreased under chemical fertilizer application, and *coxS* was also influenced by maize straw return. It indicated that chemical N fertilizer rather than maize straw imposed significant effects on CO oxidation in soil. It is consistent with the dynamic pattern of functional genes related with the multiple systems and 3 HP cycle. The N and NPK treatments had higher functional gene abundance that was related to rTCA cycle (including *korA*, *korB*, and *IDH1*) than S and PKS treatments, and the relative abundance of *PCCA*, *PCCB*, *accA*, *accC*, and *accD* related to multiple systems in C fixation was also markedly enhanced by the N, NPK and NPKS treatments. These results indicated that chemical fertilizer contributed more to the variations in soil microbial C fixation than maize straw.

The functional genes related to C degradation mostly participated in hemicellulose, starch and cellulose degradation. The abundance of functional genes related to hemicellulose, cellulose and starch degradation were significantly different from each other under different fertilization treatments in this study. The amendment of maize straw could enhance the nutrient availability for microbial growth such as organic C and N in soil, and improve the activity soil hydrolytic enzymes, and provide more C source for soil microorganisms (Zhu et al., 2019; Wu et al., 2020). In addition, previous findings showed that maize straw addition increased the amount of macroaggregates in soil, thus improving the permeability of soil and promoting the growth and development of aerobic microorganisms (Zhang et al., 2022). Therefore, the abundance of functional genes related to C degradation was higher under S, PKS and NPKS treatments than N and NPK treatments. Moreover, chemical fertilizer application decreased the abundance of functional genes related to hemicellulose degradation (i.e., *xylA*, *xylH, xylF*, and *rfbB*). It was because that long-term application of chemical fertilizer resulted in the decrease of soil microbial diversity and the change in microbial community structure, thus reducing the capacity of microbial C decomposition in soil (Jin et al., 2022). Previous study concluded that the combined application of chemical fertilizer and maize straw improved the activity of soil enzymes that degraded cellulose and hemicellulose from straw into glucose (β-Cellobiohydrolase, βxylosidase and β-glucosidase; Liu et al., 2022). Consistently, in this study, the combined application of chemical fertilizer and maize straw increased the abundance of *celF*, a functional gene related to cellulose degradation.

The abundance of functional gene *mttB* which was the highest abundance gene for methanogenesis, and decreased by 0.49, 0.69, and 0.43% under N, NPK and NPKS treatments, respectively, which was consistent with the findings of Wu P. et al. (2023). Compared with S and PKS treatments (without chemical N fertilizer), N, NPK, and NPKS treatments produced lower CH_4_. Previous study concluded that the limiting factor of CH_4_ emission was the precursor for CH_4_ production instead of the mineral nitrogen in soil (Wang Xiaoqi et al., 2017). The decomposition of maize straw increases the content of SOC, which provides a higher level of C source for soil microorganisms (Liang et al., 2021; Ren et al., 2023). Consequently, the decomposition of organic C into monosaccharides and then acids by microorganisms was accelerated, which promotes the CH_4_ production (Wang Xiaoqi et al., 2017; Zhang and Zhang, 2020). In this study, long-term fertilizer application significantly affected the abundance of CH_4_ oxidation genes including *pmoA* and *pmoB*. In conclusion, chemical fertilizer application rather than maize straw addition exhibited significant influences on soil C fixation, degradation and CH_4_ metabolism. The above results indicated that long-term fertilization affect C cycle by alter functional genes related to C cycle.

### 4.3. Correlations between functional genes and soil chemical properties

Long-term chemical fertilization can greatly alter the microbial function by directly increasing available nutrients or indirectly changing the soil physical and chemical properties. The chemical properties including SOC, DOC, ROC, TN, NO_3_^−^-N, AK, AP, TP, and pH changed significantly under different fertilization treatments in the maize rhizosphere soil in this study (Supplementary Table S1), which resultantly influenced the C fixation and degradation (Figures 4B, 5). Carbon and N sources in soil such as SOC, ROC, DOC, and TN and NO_3_^−^-N provided C and N source for microbial growth (Xie et al., 2012). Labile SOC had negative effects on C fixation and positive effects on C degradation. However, the effects of NO_3_^−^-N on C fixation and degradation were opposite with that of SOC (Figure 5). Previous studies confirmed that soil P was the most influential driver of C cycle, and could efficiently maintain the microbial growth and enhance the microbial function for mediating soil C cycle (Yao et al., 2018; Hu et al., 2022). In this study, AP had significantly positive correlations with the abundance of *bbcA*, *accD6*, *xylH*, *xylA*, *chi*, *celF*, *cbhA*, *pgl*, *malZ*, and negative correlations with that of *por*, *porB* and *coxL*. Soil pH was also an important factor regulating the soil C cycle. Soil pH could alter the availability of soluble nutrients in soil, and directly affect the contents of dissolved CO_2_ and the use of C sources (CO_2_ and HCO_3_^−^) by soil microorganisms, thereby imposing a significant influence on the C cycle (Huang et al., 2022). The result also confirmed that long-term fertilization can affect soil functional genes and carbon cycle by changing soil physical and chemical properties.

## 5. Conclusion

In this study, there were eight C fixation pathways in maize rhizosphere soil, and the dominant one was the reductive citrate cycle (rTCA cycle), followed by the dicarboxylate-hydroxybutyrate (DC/4-HB cycle), 3-hydroxypropionate bicycle (3-HP cycle) and the Calvin cycle. Long-term fertilizer application had a significant effect on soil C fixation except for the Calvin and 3-HP cycles. Chemical fertilizers increased the abundance of the C_4_-Dicarboxylic acid cycle metabolic pathway and the CAM light metabolic pathway, and inhibited the DC/4-HB cycle. Compared with CK treatment, S and PKS treatments had no significant influences on the soil C fixation. The relative abundance of CH_4_-producing metabolic pathways that used acetate as a substrate was the highest in maize rhizosphere soils, and increased significantly under N fertilizer application relative to CK. However, long-term fertilizer application had no significant effect on CH_4_ metabolic pathway that used CO_2_ as a substrate. The functional genes related to C degradation mostly participated in hemicellulose, cellulose and starch degradation. The abundance of functional genes related to C degradation was higher under S, PKS and NPKS treatments than N and NPK treatments, indicating that chemical fertilizer application had a significant influence on C degradation. In addition, chemical fertilizer application decreased the abundance of methanogenesis genes (i.e., *mttB* and *mttC*) and CH_4_ oxidation genes (i.e., *pmoA* and *pmoB*). In conclusion, this study implied that N fertilizer rather than maize straw application had significant impacts on the C cycle in maize rhizosphere soil. Moreover, soil chemical properties had significant influences on the abundance of functional genes related to C fixation and degradation, with SOC and NO_3_^−^-N being the major drivers. Overall, our results highlighted the important of long-term fertilization in C cycle and the microorganism regulated C cycle in maize rhizosphere.

## Data availability statement

The original contributions presented in the study are included in the article/Supplementary material, further inquiries can be directed to the corresponding authors.

## Author contributions

SL and WH: conceptualization and funding acquisition. CW and HC: methodology. YL and YZ: software. SL: resources and project administration. YL: data curation, writing—original draft preparation, and visualization. YL and SL: writing—review and editing. CW: supervision. All authors contributed to the article and approved the submitted version.

## Funding

This work is a contribution to Natural Science Foundation of Jilin Province of China (20210101100JC) and Special Project on Science and Technology Innovation Capacity Construction of Beijing Academy of Agriculture and Forestry Sciences (KJCX20220416).

## Conflict of interest

The authors declare that the research was conducted in the absence of any commercial or financial relationships that could be construed as a potential conflict of interest.

## Publisher’s note

All claims expressed in this article are solely those of the authors and do not necessarily represent those of their affiliated organizations, or those of the publisher, the editors and the reviewers. Any product that may be evaluated in this article, or claim that may be made by its manufacturer, is not guaranteed or endorsed by the publisher.

## Footnotes

^1^ http://exon.gatech.edu/GeneMark/

^2^ http://www.genome.jp/kegg/

^3^ https://www.genescloud.cn

^4^ https://cloud.majorbio.com/page/tools/
