# Supplementary material for: Metagenomics reveals the effect of long-term fertilization on carbon cycle in the maize rhizosphere
Source: Front Microbiol. 2023 May 19;14:1170214. doi: 10.3389/fmicb.2023.1170214 (PMC10235612; doi:10.3389/fmicb.2023.1170214)
Supplement: Supplementary file 1 [file Data_Sheet_1.docx]

Supplementary Material

Metagenomics Reveals the Effect of Long-term Fertilization on Carbon Cycle in the Maize Rhizosphere

Yanan Li^1,2^, Chengyu Wang^1,2^, Hongyan Chang^1,2^, Yumang Zhang^1,2^, Shuxia Liu^1,2*^, Wentian He^3^*

*** Correspondence: Shuxia Liu: liushuxia69@163.com;** **Wentian he: wentian_he@hotmail.com**

# Supplementary Figures and Tables

## Supplementary Figures





Fig S1. The number of genes (KO) for all metabolic pathways in CO_2_ fixation are annotated from the KEGG database at the module level in maize rhizosphere soils different fertilizers application.

Notes: the reductive pentose phosphate cycle (M00165, Calvin cycle; M00166, ribulose-5P => glyceraldehyde-3P; M00167, glyceraldehyde-3P => ribulose-5P), CAM cycle (M00168, dark; M00169, light), C4-Dicarboxylic acid cycle (M00170, M00171, M00172), rTCA cycle (M00173), DC/4-HB cycle (M00374), 3-HP/4-HB cycle (M00375), 3-HP cycle (M00376), and Wood-Ljungdahl pathway (M00377)





Fig S2. The number of genes (KO) for all metabolic pathways in CO_2_ fixation are annotated from the KEGG database at the module level in maize rhizosphere soils different fertilizers application.

Notes: Methane oxidation: M00174, Methanaogenesis: CO_2_ => methane (M00567), methanol => methane (M00356), acetate => methane (M00357), methylamine/dimethylamine/trimethylamine => methane (M00563).


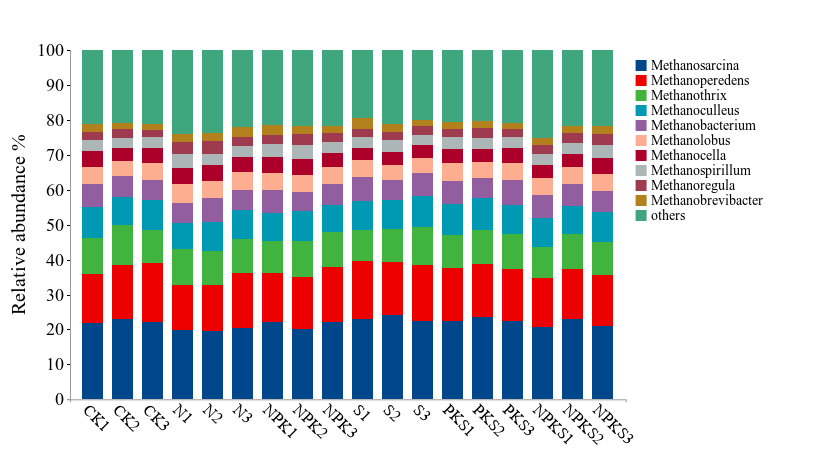
Fig S3. The relative of the top 10 Archaea genus under long-term fertilizers application under in maize rhizosphere soil.

## Supplementary Tables

# Table S1. The chemical properties of maize rhizosphere soil under long-term fertilizer application

|  | SOC g/kg | DOC mg/kg | ROC g/kg | | pH | | TN g/kg | TP g/kg |
| --- | --- | --- | --- | --- | --- | --- | --- | --- |
| CK | 18.77±0.42c | 90.99±6.20c | 3.92±0.06d | | 7.48±0.06a | | 1.22±0.00f | 6.20±0.10c |
| N | 16.50±0.27d | 95.54±9.65c | 3.65±0.19d | | 6.37±0.16c | | 1.27±0.01e | 6.00±0.08cd |
| NPK | 18.68±0.00c | 177.43±5.79b | 4.95±0.09c | | 6.77±0.14bc | | 1.36±0.00d | 9.92±0.33b |
| S | 19.84±0.31b | 181.54±8.27b | 4.89±0.06c | | 6.97±0.52b | | 1.56±0.02a | 10.19±0.29b |
| PKS | 19.46±0.20b | 195.62±15.09b | 5.47±0.14b | | 7.11±0.10ab | | 1.46±0.00c | 5.29±0.97d |
| NPKS | 21.28±0.23a | 241.12±17.55a | 6.22±0.11a | | 7.17±0.13ab | | 1.49±0.01b | 11.27±0.22a |
|  | TK g/kg | NH_4_^+^-N mg/kg | | NO_3_^-^-N mg/kg | | AP mg/kg | AK mg/kg |  |
| CK | 5.08±0.15a | 1.78±0.27b | | 0.74±0.12c | | 3.07±0.38c | 211.69±4.01d |  |
| N | 4.93±0.46a | 2.46±0.23a | | 1.48±0.36a | | 2.42±0.41c | 156.37±2.50f |  |
| NPK | 5.03±0.59a | 2.22±0.45ab | | 1.56±0.55a | | 19.93±2.14b | 187.70±4.24e |  |
| S | 5.15±0.09a | 2.35±0.46ab | | 0.60±0.25b | | 23.40±2.19a | 249.68±3.07c |  |
| PKS | 5.05±0.88a | 2.15±0.06ab | | 0.57±0.09c | | 4.20±1.22c | 303.23±8.91b |  |
| NPKS | 5.20±0.05a | 1.92±0.35ab | | 0.66±0.08c | | 23.15±0.80a | 325.39±5.79a |  |

Notes: SOC: soil organic carbon, DOC: dissolved organic carbon, ROC: readily oxidizable organic carbon, TN: total nitrogen, TP: total phosphorus, TK: total potassium, NH_4_^+^-N: Ammonium nitrogen, NO_3_^-^-N: nitrate nitrogen, AP: active phosphorus, AK: active potassium.

Table S2 the statistical table of sequencing data

|  | Reads | Counting bases (bp) | Contig.fa | N0 (%） | GC（%） | Q20（%） | Q30（%） |
| --- | --- | --- | --- | --- | --- | --- | --- |
| CK1 | 93336740 | 13989151120 | 3025467 | 0.00131 | 63.84 | 97.5 | 93.42 |
| CK2 | 90184394 | 13517390198 | 2884632 | 0.00132 | 63.78 | 97.5 | 93.4 |
| CK3 | 89394794 | 13398707236 | 2873286 | 0.00132 | 63.69 | 97.44 | 93.28 |
| N1 | 78209020 | 11731353000 | 1851993 | 0.00319 | 63.26 | 96.51 | 91.16 |
| N2 | 75133192 | 11269978800 | 1576216 | 0.00315 | 63.00 | 96.51 | 91.16 |
| N3 | 125680022 | 18852003300 | 2799788 | 0.0031 | 63.09 | 96.46 | 91.06 |
| NPK1 | 80238892 | 12025384734 | 2845519 | 0.00132 | 63.86 | 97.54 | 93.48 |
| NPK2 | 80386530 | 12047721690 | 2697026 | 0.00132 | 63.92 | 97.53 | 93.45 |
| NPK3 | 86894456 | 13024047000 | 2959256 | 0.00131 | 63.59 | 97.53 | 93.46 |
| S1 | 106586704 | 15975803212 | 3631574 | 0.0013 | 64.40 | 97.47 | 93.33 |
| S2 | 85806224 | 12861234260 | 3631574 | 0.00171 | 64.29 | 97.55 | 93.41 |
| S3 | 78428180 | 11755047386 | 2817618 | 0.00172 | 63.95 | 97.53 | 93.37 |
| PKS1 | 80870838 | 12120907500 | 2636432 | 0.00173 | 64.02 | 97.56 | 93.44 |
| PKS2 | 83000794 | 12439965728 | 2733801 | 0.00174 | 64.21 | 97.56 | 93.41 |
| PKS3 | 80907310 | 12126683472 | 2627153 | 0.00171 | 63.98 | 97.35 | 92.99 |
| NPKS1 | 94096794 | 14103651114 | 3154768 | 0.00131 | 64.19 | 97.51 | 93.42 |
| NPKS2 | 102120528 | 15306179650 | 3469531 | 0.00132 | 64.77 | 97.54 | 93.48 |
| NPKS3 | 94400800 | 14148718252 | 3203163 | 0.00132 | 63.80 | 97.44 | 93.26 |

Notes: Clean Reads: the number of pairs end sequences is counted in four rows and one unit after filtering; Counting bases: total base sequencing data volume; Contig.fa: contigs sequences were retained after filtering and removing chimeras; N0%: the percentage of fuzzy bases in total bases; Q20: percentage of bases with mass value greater than or equal to 20; Q30: percentage of bases with mass value greater than or equal to 30 i; GC: percentage of G and C type bases.

Table S3 Information of microbial functional genes involved in the C cycling processes identified in this study.

|  | Gene name | KEGG orthology number | Encoded protein [EC] |
| --- | --- | --- | --- |
| methanogeneiss | mtbA | K14082 | [methyl-Co(III) methylamine-specific corrinoid protein]:coenzyme M methyltransferase [EC:2.1.1.247] |
|  | mttB | K14083 | trimethylamine methyltransferase [EC:2.1.1.250] |
|  | mttC | K14084 | trimethylamine corrinoid protein |
|  | mtrA | k00577 | N5-methyltetrahydromethanopterin-coenzyme M methyltransferase subunit A [EC:2.1.1.86] |
| CH_4_ oxidation | pmoA | k10944 | methane monooxygenase subunit A [EC:1.14.18.3, 1.14.99.39] |
|  | pmoB | k10945 | methane monooxygenase subunit B |
|  | pmoC | k10946 | methane monooxygenase subunit C |
|  | mmoX | K16157 | methane monooxygenase component A alpha chain [EC:1.14.13.25] |
|  | mmoY | K16158 | methane monooxygenase component A beta chain [EC:1.14.13.25] |
|  | mmoZ | K16159 | methane monooxygenase component A gamma chain [EC:1.14.13.25] |
|  | mmoB | K16160 | methane monooxygenase regulatory protein B |
|  | mmoC | K16161 | methane monooxygenase component C [EC:1.14.13.25] |
| CO oxidation | coxS | k02518 | carbon monoxide dehydrogenase small subunit [EC:1.2.5.3] |
|  | coxM | k03519 | carbon monoxide dehydrogenase medium subunit [EC:1.2.5.3] |
|  | coxL | k03520 | carbon monoxide dehydrogenase large subunit [EC:1.2.5.3] |
| Calvin cycle | cbbL | k01601 | ribulose bisphosphate carboxylase large chain [EC:4.1.1.39] |
|  | cbbs | k01602 | ruBisCO small subunit [EC:4.1.1.39] |
| reductive acetyl-CoA pathway | fhs | k01938 | formyltetrahydrofolate synthetase [EC:6.3.4.3] |
|  | cooF | K00196 | anaerobic carbon-monoxide dehydrogenase iron sulfur subunit |
|  | cooC | k07321 | carbon monoxide dehydrogenase accessory protei |
| rTCA cycle | korA | k00174 | 2-oxoglutarate ferredoxin oxidoreductase subunit alpha [EC:1.2.7.11] |
|  | korB | k00175 | 2-oxoglutarate ferredoxin oxidoreductase subunit beta [EC:1.2.7.11] |
|  | korD | K00176 | 2-oxoglutarate ferredoxin oxidoreductase subunit delta [EC:1.2.7.3] |
|  | korC | K00177 | 2-oxoglutarate ferredoxin oxidoreductase subunit gamma [EC:1.2.7.3] |
|  | IDH1 | k00031 | isocitrate dehydrogenase [EC:1.1.1.42] |
| multiple systems | bccA | k11263 | propionyl-CoA carboxylase [EC:6.4.1.3] |
|  | PCCA | K01965 | propionyl-CoA carboxylase alpha chain [EC:6.4.1.3] |
|  | PCCB | K01966 | propionyl-CoA carboxylase beta chain [EC:6.4.1.3 2.1.3.15] |
|  | accD6 | K18472 | acetyl-CoA/propionyl-CoA carboxylase carboxyl transferase subunit [EC:6.4.1.2 6.4.1.3 2.1.3.15] |
|  | pccB | K19312 | acetyl-CoA/propionyl-CoA carboxylase carboxyl transferase subunit [EC:6.4.1.2 6.4.1.3 2.1.3.15] |
|  | accA | k01962 | acetyl-CoA carboxylase carboxyl transferase subunit alpha [EC:6.4.1.2, 2.1.3.15] |
|  | accC | K01961 | acetyl-CoA carboxylase, biotin carboxylase subunit [EC:6.4.1.2 6.3.4.14] |
|  | accD | K01963 | acetyl-CoA carboxylase carboxyl transferase subunit beta [EC:6.4.1.2 2.1.3.15] |
|  | facA | k01895 | acetyl-CoA synthetase [EC:6.2.1.1] |
| 3-Hydroxypropionate bicycle | porA | K00169 | pyruvate ferredoxin oxidoreductase alpha subunit [EC:1.2.7.1] |
|  | porB | K00170 | pyruvate ferredoxin oxidoreductase beta subunit [EC:1.2.7.1] |
|  | porD | K00171 | pyruvate ferredoxin oxidoreductase delta subunit [EC:1.2.7.1] |
|  | porC | K00172 | pyruvate ferredoxin oxidoreductase gamma subunit [EC:1.2.7.1] |
|  | vorG | K00189 | 2-oxoisovalerate/pyruvate ferredoxin oxidoreductase gamma subunit [EC:1.2.7.7 1.2.7.1] |
|  | por | K03737 | pyruvate-ferredoxin/flavodoxin oxidoreductase [EC:1.2.7.1 1.2.7.-] |
| Dicarboxylate-hydroxybutyrate cycle | pcc | K01595 | phosphoenolpyruvate carboxylase [EC:4.1.1.31] |
| hemicellulose | xylF | K10543 | D-xylose transporter |
|  | xylH | K10544 | D-xylose transport system permease protein |
|  | xylA | K01805 | xylose isomerase [EC:5.3.1.5] |
|  | rfbB | K01710 | phosphomannomutase [EC:4.2.1.46] |
| chition | chi | K01183 | chitinase [EC:3.2.1.14] |
| cellulose | celF | K01179 | endoglucanase F [EC:3.2.1.4] |
|  | cbhA | k19668 | 1,4-beta-cellobiohydrolase A [EC:3.2.1.91] |
| pectin | pgl | K01184 | Polygalacturonase [EC:3.2.1.15] |
|  | pel | K01728 | pectate lyase [EC:4.2.2.2] |
| starch | malZ | K01187 | alpha-glucosidase [EC:3.2.1.20] |
|  | malQ | K00705 | 4-alpha-glucanotransferase [EC:2.4.1.25] |
|  | iam | k01214 | isoamylase [EC:3.2.1.68] |

Note: KEGG, Kyoto Encyclopedia of Genes and Genomes. EC, Enzyme classification.

|  | RDA1 | RDA2 | r2 | Pr(>r) |
| --- | --- | --- | --- | --- |
| **SOC** | 0.286167 | -0.95818 | 0.793179 | **0.001** |
| **DOC** | 0.60466 | -0.79648 | 0.487528 | **0.015** |
| **ROC** | 0.551607 | -0.8341 | 0.584845 | **0.005** |
| **pH** | -0.13343 | -0.99106 | 0.420753 | **0.013** |
| TN | 0.341974 | -0.93971 | 0.343302 | 0.056 |
| **AP** | 0.837337 | -0.54669 | 0.52658 | **0.002** |
| **AK** | -0.23253 | -0.97259 | 0.678698 | **0.001** |
| **TP** | 0.867812 | -0.49689 | 0.448069 | **0.013** |
| TK | 0.34184 | -0.93976 | 0.058368 | 0.655 |
| NH_4_^+^-N | 0.374492 | 0.92723 | 0.120119 | 0.355 |
| **NO_3_^-^-N** | 0.338095 | 0.941112 | 0.637463 | **0.001** |

Table S4. The correlation between soil chemical properties and functional genes related to carbon cycle.

Notes: SOC: soil organic carbon, DOC: dissolved organic carbon, ROC: readily oxidizable organic carbon, TN: total nitrogen, TP: total phosphorus, TK: total potassium, NH_4_^+^-N: Ammonium nitrogen, NO_3_^-^-N: nitrate nitrogen, AP: active phosphorus, AK: active potassium.
